# Clinicopathological and prognostic significance of platelet to lymphocyte ratio in patients with gastric cancer

**DOI:** 10.18632/oncotarget.10490

**Published:** 2016-07-08

**Authors:** Xiaobin Gu, Xian-Shu Gao, Ming Cui, Mu Xie, Chuan Peng, Yun Bai, Wei Guo, Linjun Han, Xiaodong Gu, Wei Xiong

**Affiliations:** ^1^ Department of Radiation Oncology, Peking University First Hospital, Peking University, Beijing, China; ^2^ Graduate School of Medicine, Hebei North University, Zhangjiakou, Hebei, China; ^3^ Department of Breast Cancer Radiotherapy, Tumor Hospital of Shanxi Provence, Taiyuan, China; ^4^ Department of Oncology, Tangshan People's Hospital, Hebei, China

**Keywords:** PLR, gastric cancer, biomarker, prognosis, meta-analysis

## Abstract

The present study was aim to investigate the prognostic role of platelet to lymphocyte ratio (PLR) for patients with gastric cancer (GC) using meta-analysis. A total of 13 studies (14 cohorts) with 6,280 subjects were included. By pooling hazard ratios (HRs) and 95% confidence intervals (CIs) and odds ratios (ORs) and 95% CIs from each study, we found that elevated PLR was significantly associated with poorer overall survival (OS) (HR: 1.3, 95% CI: 1.1–1.52, *p* = 0.001; *I*^2^ = 68.5%, P_h_ < 0.001) but not with poor disease-free survival (DFS) (HR: 1.6, 95% CI: 0.88–2.9, *p* = 0.122; *I*^2^ = 87.8%, P_h_ < 0.001). Subgroup analysis showed that a high PLR significantly predicted poor OS in Caucasian populations, patients receiving chemotherapy and patients at advanced stage. In addition, the cut-off value of PLR > 160 showed adequately prognostic value. Furthermore, elevated PLR was associated with lymph node metastasis and CEA levels in GC. Our meta-analysis showed that elevated PLR could be a significant prognostic biomarker for poor OS in patients with GC.

## INTRODUCTION

During the past several decades, although gastric cancer (GC) incidence rates have been declining in most Western countries [1, 2], GC still ranks fourth in incidence among all cancers and is the third leading cause of cancer-related deaths worldwide [3]. GC at early stages is often asymptomatic and most patients are diagnosed when the disease has already advanced. A variety of prognostic factors including tumor histological type, genetic polymorphisms and tumor stage have been reported for GC [4]. Despite of these, the prognosis of GC is poor, with the 5-year survival rate being 20% [5], therefore, more novel and easily available prognostic biomarkers are needed.

The inflammatory responses to cancer have been recognized more than one century ago [6]. In recent years, clear evidence showed that inflammation plays pivotal roles in carcinogenesis and tumor metastasis [7, 8]. Laboratory parameters which reflect the status of systemic inflammation, have been investigated as prognostic biomarkers in various cancers. These inflammatory markers include modified Glasgow Prognostic Score (mGPS), neutrophil to lymphocyte ratio (NLR) and platelet to lymphocyte ratio (PLR) [9, 10]. PLR has been suggested as an independent prognostic factor in several solid tumors including colorectal cancer [11], non-small cell lung cancer [12], pancreatic cancer [13] and gastric cancer [14]. Previous evidence suggested that platelets played multiple roles in inflammatory processes, for example, platelets could facilitate neutrophils adhesion to endothelium through releasing chemokines and cytokines [15]. Platelets may also promote tumor progression through facilitation of neoangiogenesis, production of adhesion molecules and increase of early metastatic niches [16, 17]. In contrast, lymphocytes are known to hinder tumor cell proliferation and metastasis [18]and mediate antibody-dependent cell-mediated cytotoxicity (ADCC) effects [19]. Therefore, there is a biological rationale for using PLR, to measure the systemic host response in gastric cancer to predict clinical outcomes. Accumulated studies have reported the association between PLR and survival conditions in GC, however, the results were controversial. For example, Lee *et al.* [20], found that elevated PLR predicted poor overall survival (OS) in GC patients treated with chemotherapy. However, Jiang *et al.* [21] did not detect the prognostic value of PLR for GC patients receiving radical resection. As meta-analysis is an effectively analytic approach to pool these controversial findings, we thus conducted a meta-analysis to reveal the prognostic significance of PLR for overall survival (OS) and disease-free survival (DFS) and the associations between PLR and clinicopathological features in patients with GC.

## RESULTS

### Study characteristics

**Figure 1:**
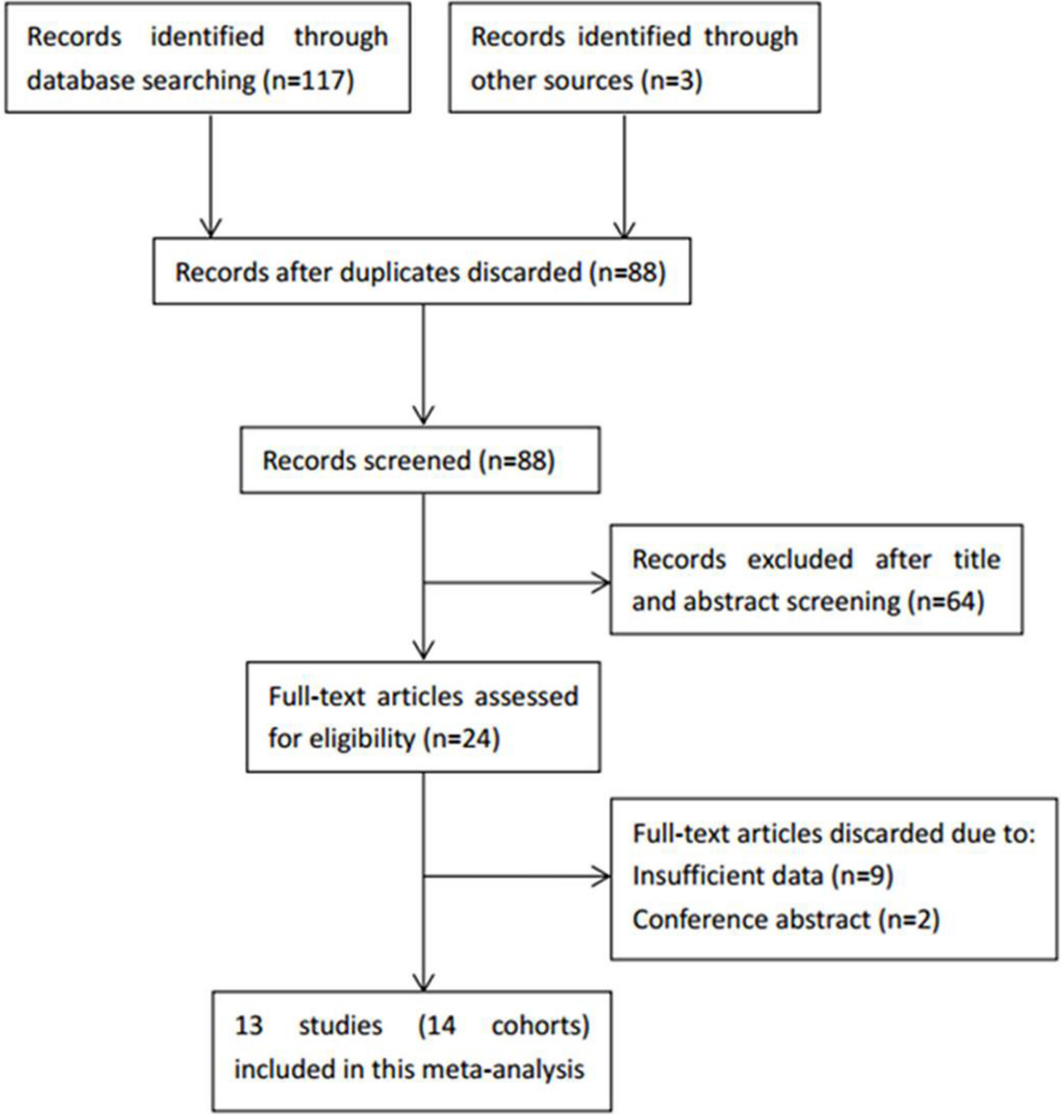
The flow diagram of publications selection

A total of 13 studies (14 cohorts) [14, 20–31] were included in the final meta-analysis. As in Aldemir's study [24], the GC patients were included as early stage and advanced stage independently, therefore, the two cohorts were extracted separately and named as Aldemir1 and Aldemir2. The selection process of the included studies was shown in Figure 1. The 14 cohorts included 6,280 GC patients. Eight cohorts [14, 21, 23, 25, 27, 29–31] were performed in China, four cohorts [22, 24, 26] were conducted in Turkey and two cohorts [20, 28] were carried out in Korea. The sample sizes ranged from 50 to 1,986. All the fourteen cohorts [14, 20–31] investigated the prognostic value of PLR for OS and three cohorts [14, 25, 31] investigated the prognostic significance of PLR for DFS. The cut-off values used by the included studies varied from 126 to 235, with a median value of PLR = 160, therefore, we selected PLR = 160 to divide the included studies in subgroup analysis. All of the studies had a NOS score > 6. The detailed information of the NOS scores of each study was shown in Supplementary Table 1. The basic characteristics of the included studies were shown in Table 1.

**Table 1:** Characteristics of included studies

Study	Year	Duration	Sample size	Follow-up (momths)	Country	Ethnicity	Treatment	Stage	Cut-off value	Survival analysis	NOS score
**Aliustaoglu**	2010	2004–2008	168	NA	Turkey	Caucasian	Chemotherapy	Advanced	160	OS	6
**Lee**	2013	2007–2010	174	14.9 (1–47.9)	Korea	Asian	Chemotherapy	Advanced	160	OS	8
**Jiang**	2014	2005–2007	377	34	China	Asian	Surgery	Early	184	OS	7
**Wang**	2014	2006–2014	439	NA	China	Asian	Mixed	Advanced	160	OS	7
**Aldemir1**	2015	2006–2013	53	NA	Turkey	Caucasian	Surgery	Early	170	OS	7
**Aldemir2**	2015	2006–2013	50	NA	Turkey	Caucasian	Chemotherapy	Advanced	170	OS	7
**Deng**	2015	2007–2009	389	24 (3–60)	China	Asian	Surgery	All	132	OS, DFS	9
**Gunaldi**	2015	NA	245	11.5	Turkey	Caucasian	Mixed	All	160	OS	7
**Hsu**	2015	2005–2011	1030	30	China	Asian	Surgery	All	132	OS	7
**Kim**	2015	2000–2009	1986	NA	Korea	Asian	Surgery	Early	126	OS	7
**Lian**	2015	2007–2010	162	60	China	Asian	Surgery	All	208	OS, DFS	8
**Liu**	2015	2005–2010	455	NA	China	Asian	Surgery	Early	188	OS	6
**Sun**	2015	1998–2008	632	55.75 (0.8–186)	China	Asian	Surgery	All	140	OS	7
**Wang**	2015	2010–2011	120	40	China	Asian	Chemotherapy	Advanced	235	OS, DFS	8

NA: not available; OS: overall survival; DFS: disease-free survival.

### PLR and OS in GC

**Figure 2:**
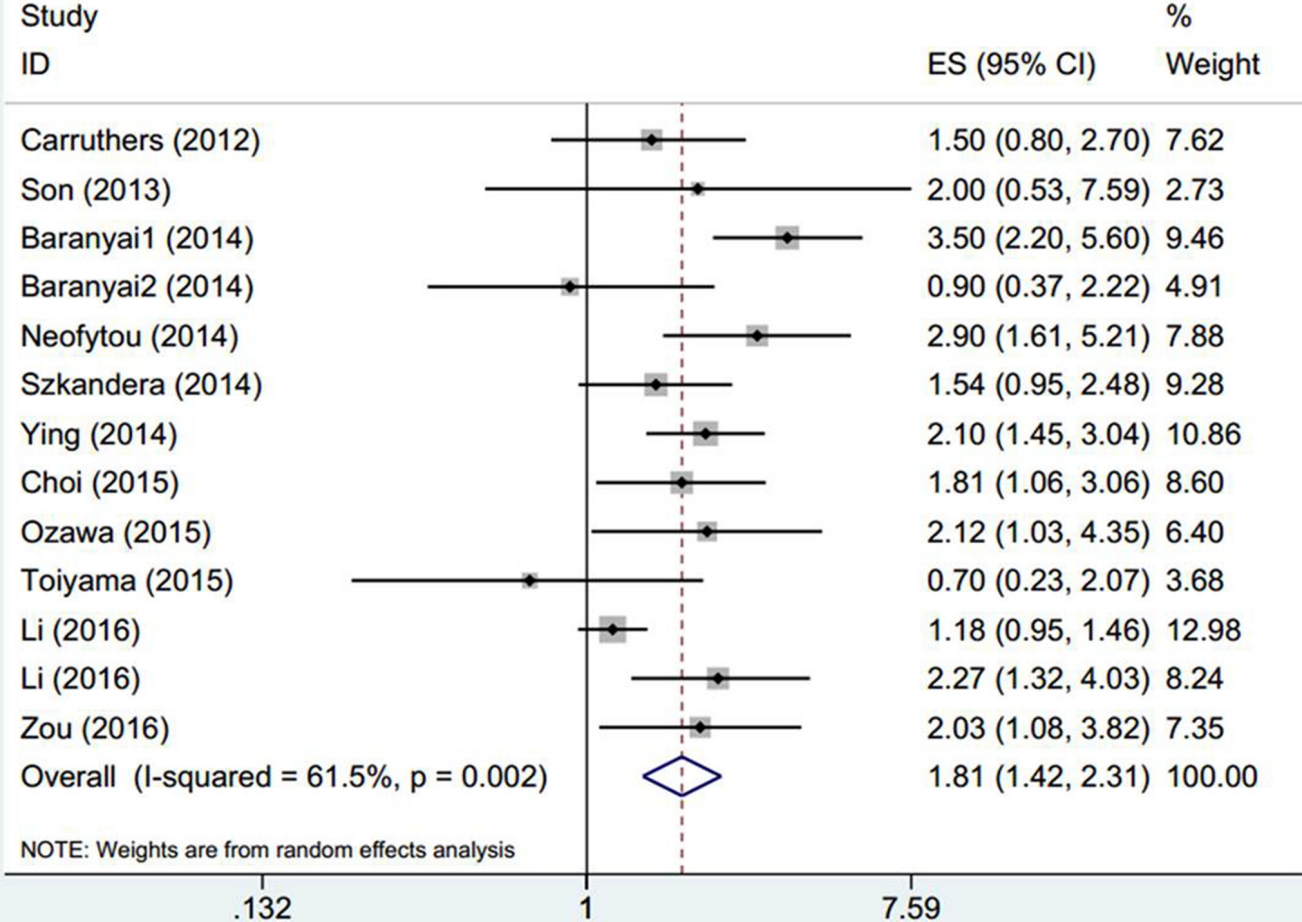
The forest plot between elevated PLR and OS in patients with GC

There were 14 cohorts with 6,280 GC patients evaluating PLR for OS (Table 2). Elevated PLR was significantly associated with poorer OS (HR: 1.3, 95% CI: 1.1–1.52, *p* = 0.001) and significant heterogeneity was observed (*I*^2^ = 68.5%, P_h_ < 0.001, Table 2, Figure 2). Subgroup analysis was conducted according to ethnicity, sample size, treatment, tumor stage and cut-off value of PLR, the results showed that elevated PLR had more significantly prognostic value for OS in Caucasian populations (HR: 1.5, 95% CI: 1.2–1.86, *p* < 0.001; *I*^2^ = 21.9%, P_h_ = 0.279). Furthermore, when stratified by treatment methods, elevated PLR significantly predicted shorter OS in patients receiving chemotherapy (HR: 1.85, 95% CI: 1.47–2.34, *p* < 0.001) with no obvious heterogeneity (*I*^2^ = 0, P_h_ = 0.923), but did not have prognostic efficiency for patients receiving mixed treatments(HR: 1.03, 95% CI: 0.68–1.58, *p* < 0.883; *I*^2^ = 77.9%, P_h_ = 0.033). Interestingly, elevated PLR indicated poor OS in patients with advanced disease, but had not value for prognostication for early disease and all tumor stages(Table 2). Of note, PLR with cut-off value > 160 still predicted poor OS for GC (HR: 1.59, 95% CI: 1.23–2.05, *p* < 0.001; *I*^2^ = 50.7%, P_h_ = 0.071), however, when PLR ≤ 160, the prognostic efficiency disappeared in the pooled results(HR: 1.15, 95% CI: 0.97–1.37, *p* = 0.113; *I*^2^ = 65.6%, P_h_ = 0.005).

**Table 2:** Main results of the meta-analysis

	Factors	No. of studies	No. of patients	Effects model	HR (95% CI)	*p*	Heterogeneity *I*^2^(%) P_h_	
OS	Overall	14	6,280	Random	1.3 (1.1–1.52)	0.001	68.5	< 0.001
	Ethnicity							
	Caucasian	4	516	Fixed	1.5 (1.2–1.86)	< 0.001	21.9	0.279
	Asian	10	5,764	Random	1.23 (1.03–1.48)	0.024	73	< 0.001
	Sample size							
	< 300	7	972	Fixed	1.66 (1.41–1.96)	< 0.001	19.8	0.279
	> 300	7	5,308	Random	1.08 (0.92–1.26)	0.35	59.6	0.021
	Treatment							
	Chemotherapy	4	512	Fixed	1.85 (1.47–2.34)	< 0.001	0	0.923
	Surgery	8	5,084	Random	1.21 (1–1.45)	0.046	66	0.004
	Mixed	2	684	Random	1.03 (0.68–1.58)	0.883	77.9	0.033
	Stage							
	Advanced	5	951	Random	1.54 (1.01–2.35)	0.045	78.6	0.001
	Early	4	2,871	Random	1.23 (0.96–1.57)	0.096	53.4	0.092
	All	5	2,458	Random	1.21 (0.95–1.54)	0.116	71.6	0.007
	Cut-off							
	≤ 160	8	5,063	Random	1.15 (0.97–1.37)	0.113	65.6	0.005
	> 160	6	1,217	Random	1.59 (1.23–2.05)	< 0.001	50.7	0.071
**DFS**	Overall	3	671	Random	1.6 (0.88–2.9)	0.122	87.8	< 0.001

P_h_: *p* value of *Q* test for heterogeneity.

### PLR and DFS in GC

**Figure 3:**
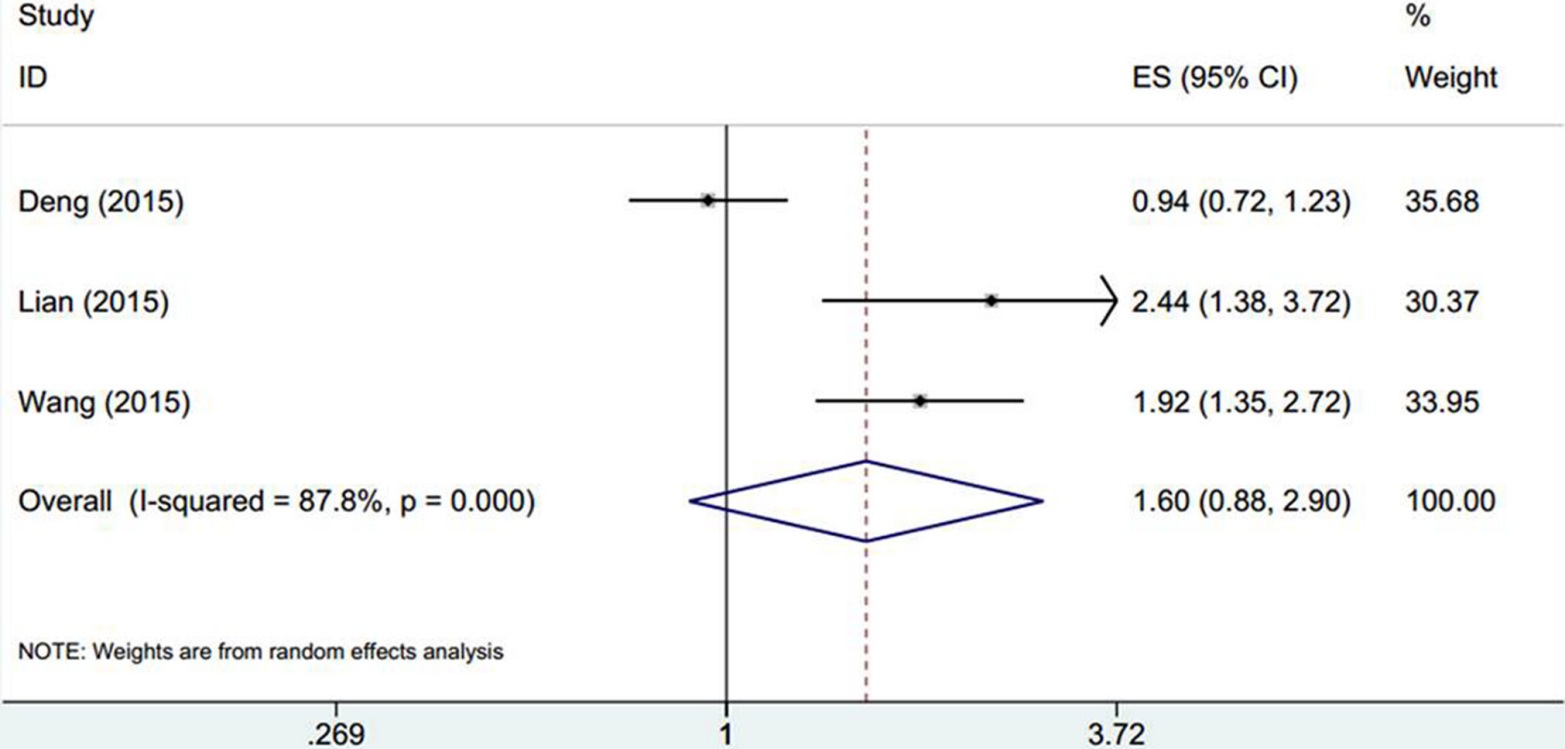
The forest plot between elevated PLR and DFS in patients with GC

Three cohorts [14, 25, 31] with 671 subjects explored the association between elevated PLR and DFS in GC. The pooled data showed that PLR had no prognostic role for DFS in GC (HR: 1.6, 95% CI: 0.88–2.9, *p* = 0.122; *I*^2^ = 87.8%, P_h_< 0.001; Table 2, Figure 3).

### PLR and tumor clinicopathological parameters

To further exploit the impact of PLR on the clinical features in GC, we identified ten clinical factors in GC and extracted the patient amounts in PLR high and PLR low groups regarding each factor. The ten factors could be classified as three categories: first, general information about patients, including gender and age; second, parameters reflecting invasiveness of tumor, including TNM stage, tumor differentiation, depth of invasion, tumor size, lymph node metastasis and distant metastasis; third, specific indexes for GC, including Lauren type, and CEA level. As shown in Table 3, the results demonstrated that high PLR was positively correlated with lymph node metastasis (*n* = 4, HR = 1.56, 95% CI: 1.33–1.83, *p* < 0.001) and CEA (*n* = 2, HR = 1.57, 95% CI: 1.11–2.24, *p* = 0.012). Whereas elevated PLR was not shown to be associated with age, gender, TNM stage, tumor differentiation, depth of invasion, tumor size, Lauren type or distant metastasis.

**Table 3:** Meta-analysis of the association between PLR and clinicopathological features of gastric cancer

Variable	No. of studies	No. of patients	OR (95% CI)	*p*	Heterogeneity *I*^2^(%) P_h_		Publication bias Begg's p
**Gender (male vs. female)**	8	3892	1.12 (0.61–2.02)	0.718	91.6	< 0.001	0.12
**Age (≥ median vs. < median)**	6	1661	1.04 (0.7–1.54)	0.847	68.3	0.008	0.851
**TNM stage (III–IV vs. I–II)**	5	3159	1.16 (0.61–2.22)	0.644	90.1	< 0.001	0.303
**Tumor differentiation (poor vs. moderate/high)**	5	3084	1.06 (0.91–1.24)	0.465	3.9	0.385	0.086
**Depth of invasion (T3–T4 vs. T1–T2)**	4	2782	1.02 (0.37–2.78)	0.972	94.4	< 0.001	0.308
**Tumor size (> 5 cm vs. < 5 cm)**	4	1098	0.91 (0.58–1.44)	0.697	54.7	0.085	0.089
**Lymph node metastasis (yes vs. no)**	4	2997	1.56 (1.33–1.83)	< 0.001	21.8	0.28	1
**Lauren type (diffuse type vs. intestinal type)**	3	456	1.04 (0.68–1.6)	0.841	0	0.569	0.602
**Distant metastasis (yes vs. no)**	3	683	0.51 (0.11–2.34)	0.387	93.8	< 0.001	0.296
**CEA (> 5 ng/ml vs. < 5 ng/ml)**	2	563	1.57 (1.11–2.24)	0.012	0	0.988	1

### Sensitivity analysis

**Figure 4:**
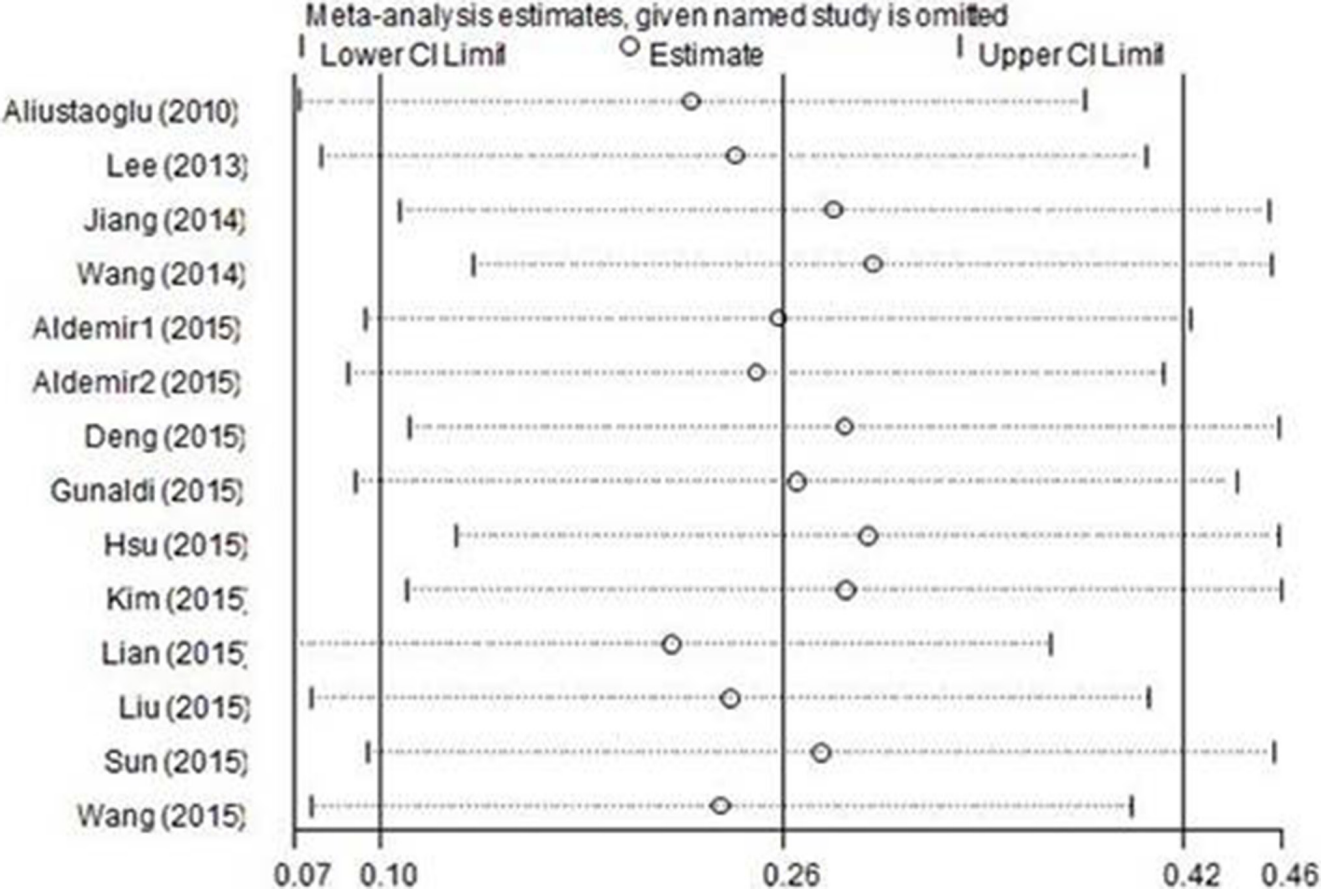
Sensitivity analysis of PLR on OS in GC patients

Every single study was moved out and thereafter the pooled data was recalculated to test the stability of the results. The results of the sensitivity analysis were shown in Figure 4. The corresponding pooled HRs did not substantially change, which confirmed the robustness of our results.

### Publication bias

**Figure 5:**
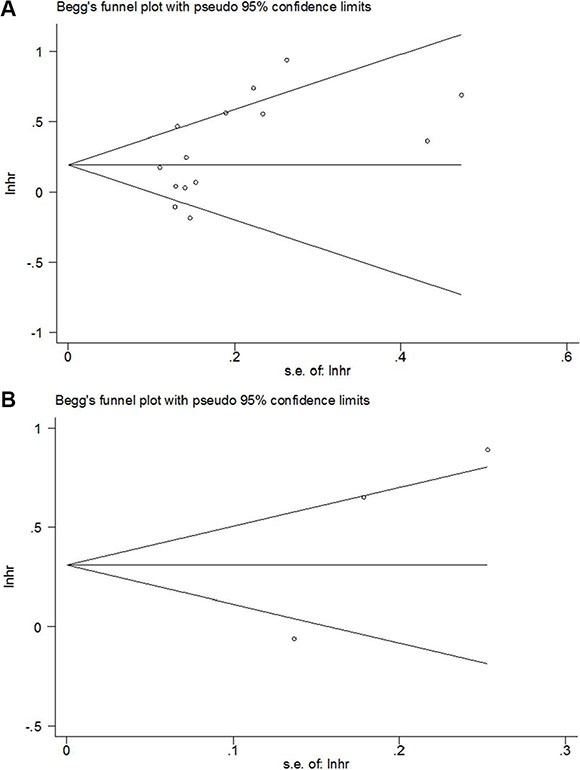
Begg's funnel plot of publication bias test for (A) OS and (B) DFS in GC

Publication bias was assessed by using Begg's test. The results indicated that there was no significant publication bias in OS and DFS (*p* = 0.08 for OS and *p* = 0.296 for DFS, respectively; Figure 5). In addition, there was also no significant publication bias for the analyses involving the relationship between PLR and clinical features in GC (Table 3).

## DISCUSSION

The current study was designed to investigate the prognostic value of elevated PLR for OS and DFS in patients with GC by meta-analysis. Pooled results from 14 cohorts with 6,280 subjects demonstrated that elevated PLR was associated with poor OS. However, PLR had not prognostic role for DFS. Moreover, elevated PLR was correlated with lymph node metastasis and CEA levels in patients with GC. To the best of our knowledge, this meta-analysis was the first to identify the prognostic role of PLR in GC.

The strong linkage between immune responses and cancer progression was increasingly investigated in the past decade. Cancer related inflammation could attenuate antitumor activity of the host through recruiting immunosuppressive cells such as regulatory T cells (Treg) [32] and myeloid-derived suppressor cells (MDSC) [33, 34]. A variety of chemokines and cytokines secreted in the tumor microenvironment could also facilitate tumor progression and metastasis [35]. The mechanisms between carcinogenesis, platelets assembly and lymphocytopenia remained unclear. Notably, recent studies demonstrated that platelet-derived signals were necessary for the recruitment of granulocytes, which could further contribute to the formation of early metastatic niches for tumor cells [17]. Moreover, platelets could also promote the communication between primary tumor cells and bone remodeling alterations prior to metastasis [36]. These could be the potential reasons for the association between elevated PLR and lymph node metastasis in the present study. In addition, lymphocytopenia and the suppression of lymphocytes activity induced by the systemic inflammatory response impairs the innate cellular immunity [37]. Thus, the combination of blood parameters such as PLR could predict prognosis more accurately. What's more, the value of PLR could be acquired from the routine laboratory tests, which provides clinical implications at a low cost.

Recently, several meta-analyses [38, 39] investigated the prognostic value of PLR in various solid tumors. In Templeton *et al*.'s work including twenty studies, they found that elevated PLR was associated with poor OS in colorectal cancer, hepatocellular carcinoma, ovarian cancer and pancreatic carcinoma [38]. Whereas, only three studies on gastric cancer were included in their meta-analysis and the limited data may restrain the credibility of the results. Another work, reported by Zhou and colleagues [39], showed that high PLR had prognostic efficiency for OS in cancer by pooling data from 26 studies with 13,964 patients. Compared with the previous studies [38, 39], the current study including 14 cohorts with 6,280 GC patients was more comprehensive with sufficient data. Moreover, we not only investigated the prognostic value of PLR for OS, but also reported the results for DFS in GC. The associations between PLR and clinicopathological characteristics were also explored. Therefore, our meta-analysis had more specificity for GC population and the adequate data made the results convincing. In the present study, we selected PLR as the study object, which was frequently compared with another blood-derived index, NLR, in included studies [14, 20, 21, 23, 25–28, 31]. NLR was also widely investigated as a prognostic indicator for GC, for it showed significant association with patients survival [20, 27]. As the present study was designed to investigate the relationship between PLR and GC, the studies regarding NLR on GC were partially included and a comprehensive conclusion could not be drew according to these studies. Therefore, the prognostic efficiency between PLR and NLR in GC could not be directly compared in this meta-analysis. Interestingly, we noted that several meta-analyses [40–43] had exploited the prognostic role of NLR in GC. The pooled HRs and 95% CIs of NLR on OS in GC ranged from HR = 1.65 (95% CI: 1.47–1.83) [42] to HR = 2.16 (95% CI: 1.86–2.51) [41], which were higher than the HR and 95% CI of PLR for OS (HR = 1.3, 95% CI: 1.1–1.52) in the present study. Previous meta-analyses [40–43] also indicated that NLR was associated with poor DFS in GC, whereas we did not find such correlation of PLR and DFS. These results suggested that NLR might have more powerful prognostic efficiency for poor OS in GC than PLR and could predict shorter DFS in GC when PLR could not. This phenomenon may be due to neutrophils, as the immune cells of the innate system, were more intensely and comprehensively involved in immune responses, compared with platelets, therefore, NLR could be more intensively influenced in GC and be more sensitive than PLR. This possible explanation should be verified in further studies.

There were some limitations need to be addressed in this meta-analysis. First, only three studies investigating the role of PLR for DFS prognostication were analyzed. The sample size was relatively small and subgroup analysis was not performed due to limited data. Second, the cut-off values of PLR were various in the studies, which calls for uniformly used value in further investigations.

In summary, we found that elevated PLR was a prognostic factor for poor OS, but not for DFS in GC patients. Furthermore, a high PLR had more significantly prognostic significance for OS in Caucasians, patients receiving chemotherapy and at advanced stage. Elevated PLR was also associated with lymph node metastasis in GC. Due to the limitations in this study, more large scale studies using uniform cut-off value of PLR are needed to validate our results.

## MATERIALS AND METHODS

### Literature search

A thorough literature searching was conducted in the databases of Pubmed, Embase and Web of Science. The last search was updated to March 2016. The search strategy was as follows: (PLR or platelet to lymphocyte ratio or platelet-lymphocyte ratio) and (gastric cancer or GC or gastric carcinoma or gastric neoplasm or stomach neoplasms). Only studies in English were included. The reference lists were manually retrieved for additional studies. Ethical approval was not required for this study because this was a meta-analysis.

### Selection criteria

Studies included in the meta-analysis need to meet the following criteria: (1) the value of PLR was acquired from a peripheral venous blood test before treatment, PLR was calculated as the ratio of the platelets to lymphocytes; (2) the diagnosis of GC was pathologically confirmed; (3) HRs and 95% CIs for PLR in OS and (or) DFS were reported, or could be calculated from raw data in the articles; (4) the cut-off value of PLR was reported. The exclusion criteria were: (1) letters, conference abstracts or review articles; (2) animal studies; (3) insufficient data to estimate HRs and 95% CIs; (4) did not present the cut-off value for elevated PLR; (5) not published in English.

### Data extraction

Based on a consensus on all items, two investigators (XB,G and XS,G) independently extracted the following information from each study: surname of the first author, year of publication, country, duration of the studies, sample size, treatment methods, stages of the disease, HRs with 95% CIs and the cut-off value of elevated PLR. Disagreement was resolved by consulting the third investigator (M,X).

### Quality assessment

The quality of included studies was assessed according to the Newcastle-Ottawa Scale (NOS)(http://www.ohri.ca/programs/clinical_epidemiology/oxford.asp) by two reviewers (W,G and M,C). The maximum score is 9 points and studies with an NOS score ≥ 6 were considered as high-quality researches.

### Statistical analysis

HRs and 95% CIs for OS and DFS were directly obtained from each study if available or were calculated from raw data using the method reported by Tierney *et al.* [44]. When analyzing the relationship between PLR and clinicopathological factors, odds ratios (OR) and 95% CI were combined. The heterogeneity between the studies was estimated with the χ^2^-based *Q* test and Higgins' *I*^2^ statistic. A *p*-value < 0.1 for the *Q*-test or *I*^2^ > 50% indicated significant heterogeneity, and the random-effects model (DerSimonian and Laird method) was used, otherwise, the fixed-effects model (Mantel-Haenszel method) was applied. Sensitivity analysis was performed by omitting each single study in turn to assess the stability of the pooled results. Begg's funnel plot was carried out to examine the publication bias. Statistical data were analyzed using STATA 12.0 (College Station, TX, USA). *P* < 0.05 was considered as statistically significant.

## SUPPLEMENTARY MATERIALS FIGURES AND TABLES




