# Supplementary material for: Clinicopathological and prognostic significance of platelet to lymphocyte ratio in patients with gastric cancer
Source: Oncotarget. 2016 Jul 8;7(31):49878–87. doi: 10.18632/oncotarget.10490 (PMC5226554; doi:10.18632/oncotarget.10490)
Supplement: Supplementary file 2 [file oncotarget-07-49878-s002.docx]

**NEWCASTLE - OTTAWA QUALITY ASSESSMENT SCALE**

**COHORT STUDIES**

Note: A study can be awarded a maximum of one star for each numbered item within the Selection and Outcome categories. A maximum of two stars can be given for Comparability

**Selection**

1) Representativeness of the exposed cohort

a) truly representative of the average GC in the community **🟑**

b) somewhat representative of the average GC in the community **🟑**

c) selected group of users eg nurses, volunteers

d) no description of the derivation of the cohort

2) Selection of the non exposed cohort

a) drawn from the same community as the exposed cohort **🟑**

b) drawn from a different source

c) no description of the derivation of the non exposed cohort

3) Ascertainment of exposure

a) secure record (eg surgical records) **🟑**

b) structured interview **🟑**

c) written self report

d) no description

4) Demonstration that outcome of interest was not present at start of study

a) yes **🟑**

b) no

**Comparability**

1) Comparability of cohorts on the basis of the design or analysis

a) study controls for OS,DFS/PFS .**🟑**

b) study controls for any additional factor Age, gender, TNM stage etc.**🟑**

**Outcome**

1) Assessment of outcome

a) independent blind assessment **🟑**

b) record linkage **🟑**

c) self report

d) no description

2) Was follow-up long enough for outcomes to occur

a) yes (3 years) **🟑**

b) no

3) Adequacy of follow up of cohorts

a) complete follow up - all subjects accounted for **🟑**

b) subjects lost to follow up unlikely to introduce bias - small number lost - > 20 % follow up, or description provided of those lost **🟑**

c) follow up rate < 80% (select an adequate %) and no description of those lost

d) no statement

Table 1.Quality Assessment of included studies based on the Newcastle-Ottawa Scales

| Study | Ref. | Representativeness of the exposed cohort  (score 0-1) | Selection of the non exposed cohort  (score 0-1) | Ascertainment of exposure  (score 0-1) | Demonstration that outcome of interest was not present at start of study  (score 0-1) | Comparability of cohorts on the basis of the design or analysis  (score 0-2) | Assessment of outcome  (score 0-1) | Was follow-up long enough for outcomes to occur  (score 0-1) | Adequacy of follow up of cohorts  (score 0-1) | Total |
| --- | --- | --- | --- | --- | --- | --- | --- | --- | --- | --- |
| Aliustaoglu |  | 1 | 1 | 1 | 1 | 1 | 1 | 0 | 0 | 6/9 |
| Lee |  | 1 | 1 | 1 | 1 | 2 | 1 | 1 | 0 | 8/9 |
| Jiang |  | 1 | 1 | 1 | 1 | 1 | 1 | 1 | 0 | 7/9 |
| Wang |  | 1 | 1 | 1 | 1 | 2 | 1 | 0 | 0 | 7/9 |
| Aldemir1 |  | 1 | 1 | 1 | 1 | 2 | 1 | 0 | 0 | 7/9 |
| Aldemir2 |  | 1 | 1 | 1 | 1 | 2 | 1 | 0 | 0 | 7/9 |
| Deng |  | 1 | 1 | 1 | 1 | 2 | 1 | 1 | 1 | 9/9 |
| Gunaldi |  | 1 | 1 | 1 | 1 | 2 | 1 | 0 | 0 | 7/9 |
| Hsu |  | 1 | 1 | 1 | 1 | 1 | 1 | 1 | 0 | 7/9 |
| Kim |  | 1 | 1 | 1 | 1 | 2 | 1 | 0 | 0 | 7/9 |
| Lian |  | 1 | 1 | 1 | 1 | 2 | 1 | 1 | 0 | 8/9 |
| Liu |  | 1 | 1 | 1 | 1 | 1 | 1 | 0 | 0 | 6/9 |
| Sun |  | 1 | 1 | 1 | 1 | 1 | 1 | 1 | 0 | 7/9 |
| Wang |  | 1 | 1 | 1 | 1 | 2 | 1 | 1 | 0 | 8/9 |
